# Associations Between Brain Structural Connectivity and 1-Year Demoralization in Breast Cancer: A Longitudinal Diffusion Tensor Imaging Study

**DOI:** 10.1155/2024/5595912

**Published:** 2024-09-26

**Authors:** Mu Zi Liang, Peng Chen, Ying Tang, Yu Yan Liang, Shu Han Li, Guang Yun Hu, Zhe Sun, Yuan Liang Yu, Alex Molassiotis, M. Tish Knobf, Zeng Jie Ye

**Affiliations:** ^1^Guangdong Academy of Population Development, Guangzhou, China; ^2^Basic Medical School, Guizhou University of Traditional Chinese Medicine, Guiyang, China; ^3^Institute of Tumor, Guangzhou University of Chinese Medicine, Guangzhou, China; ^4^Shenzhen Bao'an Traditional Chinese Medicine Hospital, Guangzhou University of Chinese Medicine, Shenzhen, China; ^5^School of Nursing, Guangzhou University of Chinese Medicine, Guangzhou, China; ^6^Army Medical University, Chongqing Municipality, China; ^7^The First Affiliated Hospital, Guangzhou University of Chinese Medicine, Guangzhou, China; ^8^South China University of Technology, Guangzhou, China; ^9^College of Arts, Humanities and Education, University of Derby, Derby, UK; ^10^School of Nursing, Yale University, Orange, Connecticut, USA; ^11^School of Nursing, Guangzhou Medical University, Guangzhou, China

**Keywords:** be resilient to breast cancer, brain structural connectivity, breast cancer, correlational tractography, demoralization, DTI

## Abstract

**Purposes:** This study aims to explore the association between brain structural connectivity and 1-year demoralization in patients with newly diagnosed breast cancer.

**Methods:** Patients were enrolled from a multicenter longitudinal program named as *Be Resilient to Breast Cancer* (BRBC) between 2017 and 2019. Brain structural connectivity was assessed with diffusion tensor imaging (DTI) at baseline and the demoralization scale II collected self-report data at baseline and 1 year later. A data-driven correlational tractography was performed to recognize significant neural pathways associated with the group membership (increased vs. nonincreased demoralization). The incremental prediction values of Quantitative Anisotropy (QA) extracted from the significant white matter tracts against the group membership were evaluated.

**Results:** 21.2% (*N* = 31) reported increased 1-year demoralization. Inferior fronto-occipital fasciculus (IFOF) was associated with 1-year demoralization in breast cancer. The incremental prediction values of QAs in net reclassification improvement (NRI) and integrated discrimination improvement (IDI) ranged from 8.11% to 46.89% and 9.12% to 23.95%, respectively, over the conventional tumor-nodal metatasis (TNM) staging model.

**Conclusion:** Anisotropy in IFOF is a potential prediction neuromarker to 1-year demoralization in patients with newly diagnosed breast cancer.

**Trial Registration:** ClinicalTrials.gov identifier: NCT03026374

## 1. Introduction

In 2022, 20 million new cancer cases are identified worldwide and breast cancer accounts for 11.6% [1]. Due to early screening and innovative anticancer treatments in breast cancer, a 5-year survival > 90% and > 80% have been achieved in western countries and China, respectively [2]. As persistent physical and psychological symptoms have been reported following therapy, breast cancer survivorship has received increasing attention from multidisciplinary researchers [3]. Demoralization, defined as loss of confidence in one's ability to cope, is associated with feelings of helplessness, hopelessness, and discouragement according to the International Classification of Diseases, 11^th^ revision (ICD-11), and has been reported across cancer and palliative care settings (ranged from 36%–52%) [4]. It can also be conceptualized as a clinical manifestation of allostatic overload and is associated with physical symptoms (i.e., fatigue, concentration), quality of life (QoL), and survival in patients with cancer [5–8]. However, it is not recognized as a distinct syndrome in American Psychiatric Association's Diagnostic and Statistical Manual of Mental Disorders, Fifth Edition, Text Revision (DSM-5-TR) and frequently confused with a depression disorder [9]. Compared to depression disorder, the literature about the neurobiological underpinnings of demoralization is relatively scarce, and it may be associated with dopamine disequilibrium in dorsolateral prefrontal cortex (DLPFC) and anterior cingulate circuits (ACC) from the perspective of molecular pathways [10, 11]. For example, psilocybin has been identified as an effective treatment for demoralization in cancer by potentially promoting neural plasticity and retaining normal functioning in brain networks, although the underlying mechanisms remain unclear [12]. However, the association between brain structural connectivity and demoralization in patients with cancer diagnoses has not been explored.

Brain structural connectivity primarily refers to the white matter tracts for signal transmission between different brain regions which are composed of axonal bundles [13]. The integrity and connectivity of these interconnected tracts can be tracked and mapped by noninvasive diffusion MRI techniques (i.e., Diffusion Tensor Imaging, DTI), and a white matter's intricate network will be invaluable to unveil the association between brain's structural framework and demoralization [14]. Thus, in the current study, DTI was utilized to map the brain's structural framework in patients with breast cancer from a multicenter longitudinal program named as *Be Resilient to Breast Cancer* (BRBC) between 2017 and 2019, and analyzed by a data-driven correlational tractography (CT) to recognize significant neural pathways associated with demoralization. In consideration of relatively stable property of white matter tracts, we were also interested whether white matter microstructure profiles at baseline could predict changes in 1-year demoralization (increased vs. nonincreased demoralization), which will help design clinical interventions (i.e., individualized meaning-centered psychotherapy, IMCP) for those at risk [15, 16]. We hypothesized that: (1) Significant neural pathways would be recognized against group membership (increased vs. nonincreased demoralization), and (2) White matter microstructure profiles could provide incremental prediction ability to 1-year demoralization.

## 2. Method

### 2.1. Sample

**Figure 1:**
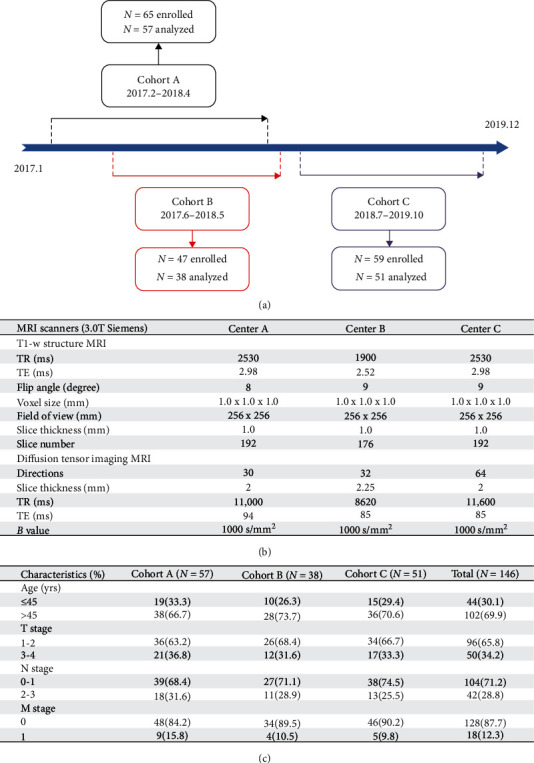
(a) Enrollment line in the *Be Resilient to Breast Cancer* (BRBC). (b) DTI parameters in different centers. (c) Demographic and clinical characteristics.

In Figure 1a, the patients with breast cancer were enrolled from our previous multicenter longitudinal program named as *Be Resilient to Breast Cancer* (BRBC) [17–21]. The inclusion criteria for patients were: (1) aged > 18 years, (2) fluent in Mandarin or Cantonese, and (3) informed consent. The exclusion criteria were: (1) life expectancy less than 12 months, (2) declined to participate in the current study, which was detailed elsewhere [22–24]. Of a sample of 171 patients, 146 (85.4%) completed the demoralization scale and DTI imaging. Three centers (cohort A, B, and C) were assigned to collect the data. DTI imaging was acquired before the anticancer treatment (T0) while demoralization data were collected at baseline (T0) and 1 year later (T1). Research nurses were trained to help facilitate the data collection. Patients were all informed consent, and ethics approval was obtained from Guangzhou University of Chinese Medicine (2016KYTD08).

### 2.2. Sample Size

According to a 0.5 standard deviation (SD) increasement in the 1-year demoralization increased group, the sample size was calculated based on the effect size of 0.5, type I error of 5% and power of 80%, achieving a least number of 128 by G*⁣*^*∗*^power software. Thus, the sample size of 146 was efficient in the current study.

### 2.3. Data Collection

#### 2.3.1. Demoralization Scale-II (DS-II)

The Chinese Version has been validated in cancer patients and consists of two dimensions including: (1) meaning and goals, and (2) stress and coping [25, 26]. A Likert-3 design is applied with higher total scores indicating higher demoralization levels. The Cronbach's coefficient was 0.87 in the current study. In addition, based on the changes in total score between T0 and T1, a 0.5 SD increasement was employed as the golden standard (increased vs., non-increased) against the predication ability of anisotropy in significant neural pathways.

#### 2.3.2. DTI Data Acquisition and Preprocessing

DTI data were acquired by using 3.0T Siemens scanners across different centers (centers A, B, and C), and the parameters for different scanners were described in Figure 1b. Echo planar imaging (EPI) was performed to collect the data and DSI Studio, using a deterministic fiber tracking algorithm, was employed to handle with eddy current distortion, subjects' movement, and other preprocessing issues [27, 28]. Q-space diffeomorphic reconstruction (QSDR) was utilized in the Montreal Neurological Institute (MNI) space with a diffusion sampling length ratio of 1.25, and followed by the calculation of QA, fractional anisotropy (FA), and radial diffusivity (RD) [29].

#### 2.3.3. Data Analysis

First, a data-driven approach named as CT was performed in DTI imaging to recognize significant neural pathways associated with the group membership (increased vs. non-increased) at the group level. It uses a bottom-up analytical approach to track the local connectivity patterns along the fiber pathways and can measure variability within core white fibers, which will be more sensitive than conventional region-of-interest (ROI) methods [30]. A 2.0 T threshold was set to determine the association after controlling the covariates of age, TNM staging, and center assignment. Filtered by topology-informed pruning with 16 iterations, a length threshold of 20 voxels with 4,000 permutations as well as a false discovery rate (FDR) < 0.05 was chosen to retain robust tracking fibers. Second, QA was extracted from those significant fibers and incorporated into a new prediction model against the 1-year demoralization to estimate the incremental values. Area under the curve (AUC), net reclassification improvement (NRI), integrated discrimination improvement (IDI), calibration and decision curves, and clinical impact curves were evaluated according to the TRIPOD guideline [31–33]. DSI Studio and MATLAB R2021b were utilized for the DTI-related analysis, and R software was used for the prediction analysis.

## 3. Results

### 3.1. Patient Demographics

From each of the three cohorts, 87.7%, 80.9%, and 86.4% of patients from BRBC completed the DTI and demoralization data collection. No significant differences were identified between the lost follow-up and patients included in the final analysis (all *P* > 0.05). Other information was detailed in Figure 1c.

### 3.2. CT and Significant Tracking Fibers

**Figure 2:**
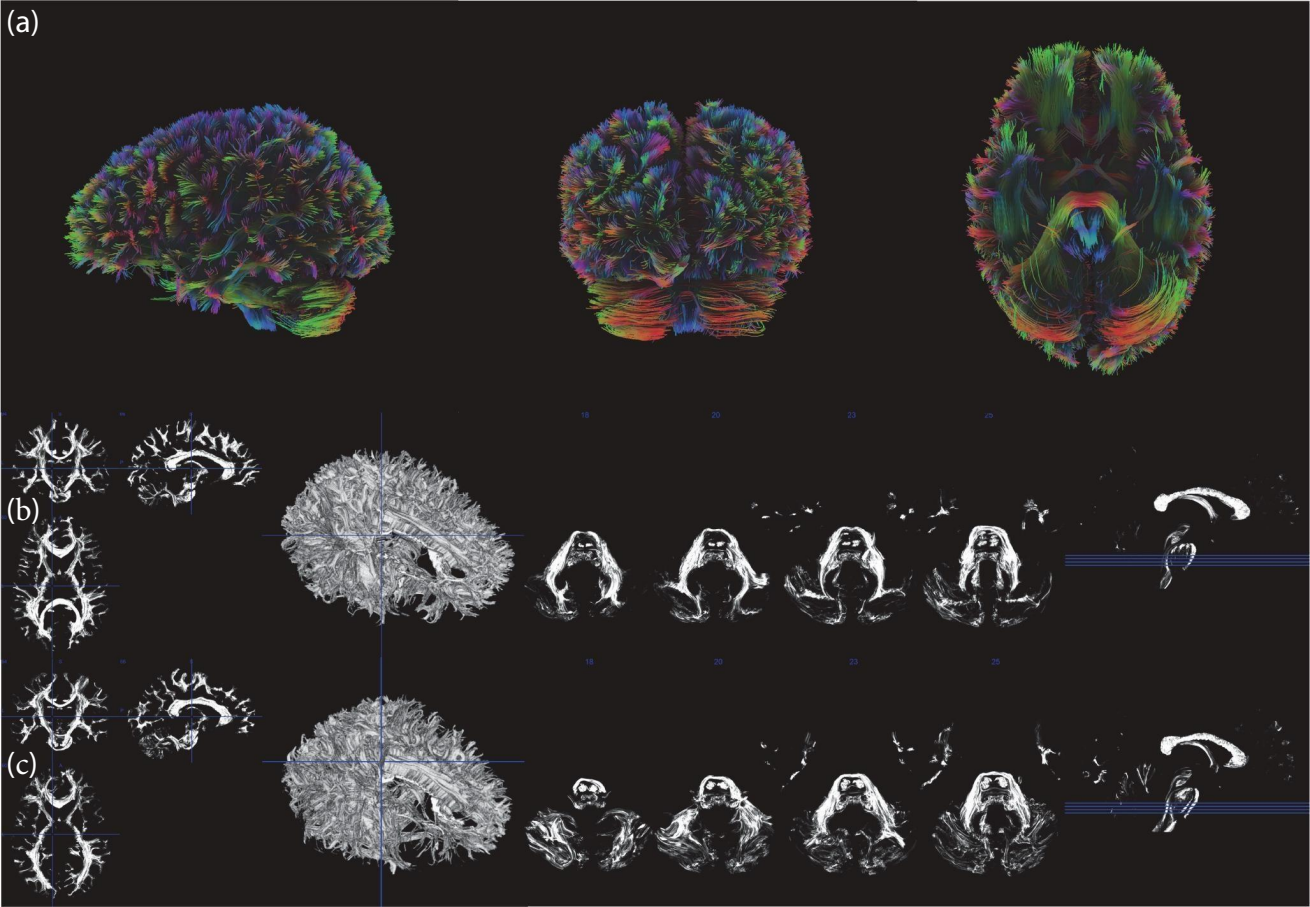
(a) Reconstruction using a deterministic fiber tracking algorithm. (b) Super-resolution white matter imaging in the 1-year increased demoralization group. (c) Super-resolution white matter imaging in the 1-year nonincreased demoralization group.

**Figure 3:**
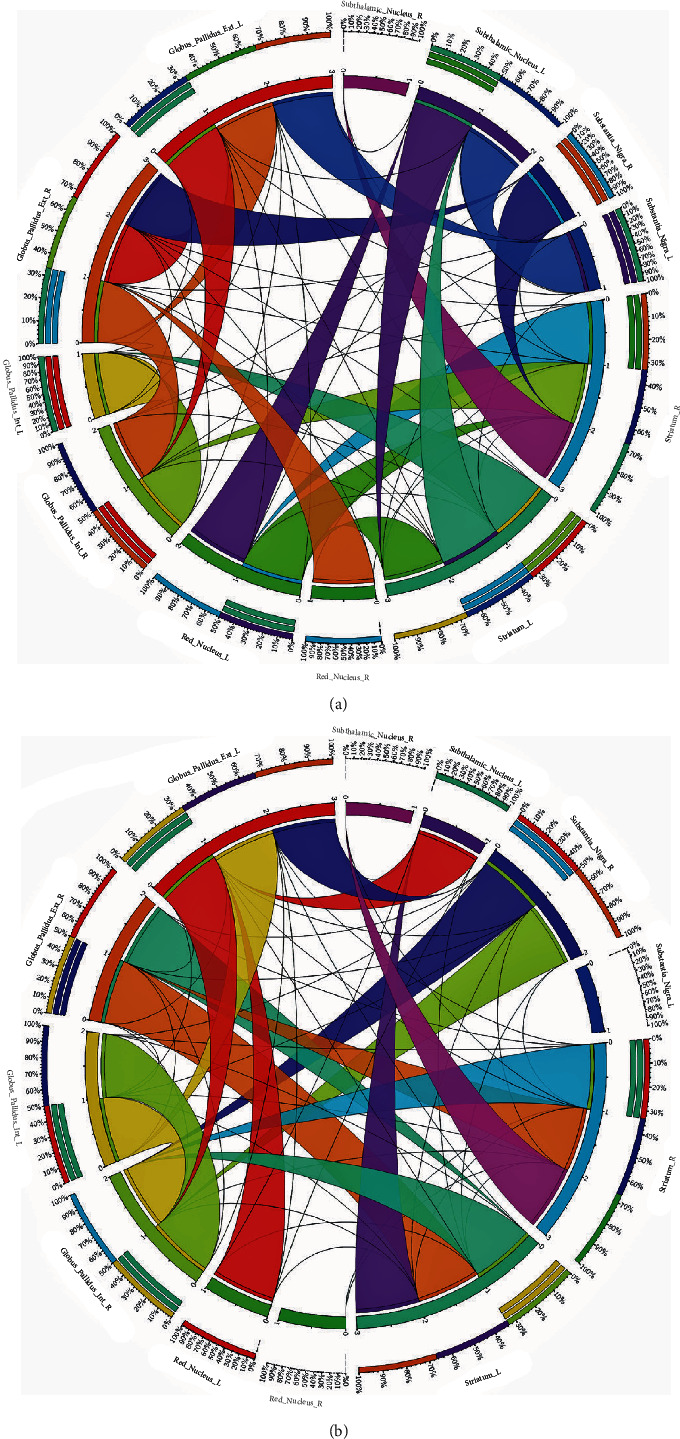
(a) Connectivity matrix in the 1-year increased demoralization group. (b) Connectivity matrix in the 1-year nonincreased demoralization group.

**Figure 4:**
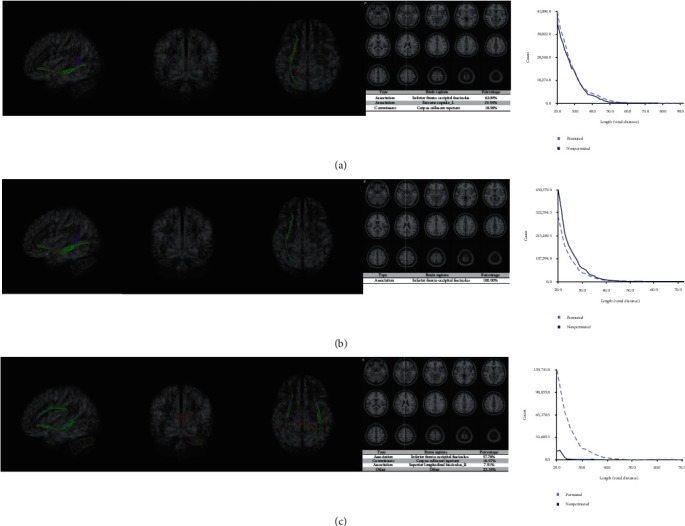
(a) Significant neural pathways recognized based on the quantitative anisotropy (QA). (b) Significant neural pathways recognized based on the fractional anisotropy (FA). (c) Significant neural pathways recognized based on the radial diffusivity (RD).

QSDR was utilized for reconstruction using a deterministic fiber tracking algorithm in all patients (Figure 2a). In Figures 2b,c, super-resolution white matter imaging for increased and nonincreased demoralization groups were detailed separately. In addition, the structural connectivity matrix in the two groups were also described in Figures 3a,b separately. In Figure 4a, significant correlations were recognized by CT between QA values and group membership in IFOF, 62.09%, extreme capsule left (19.54%), and corpus callosum tapetum (18.38%). In Figure 4b, significant correlations were recognized between FA values and group membership in IFOF (100%). In Figure 4c, significant correlations were recognized between RD values and group membership in IFOF (57.78%), corpus callosum tapetum (10.97%), and superior longitudinal fasciculus right (7.91%). Thus, in consideration of robust findings from QA, FA, and RD values, QA values were extracted from IFOF and incorporated into a new prediction model against the 1-year demoralization to estimate the incremental values.

### 3.3. The Incremental Prediction Ability of Brain Connectomics

**Figure 5:**
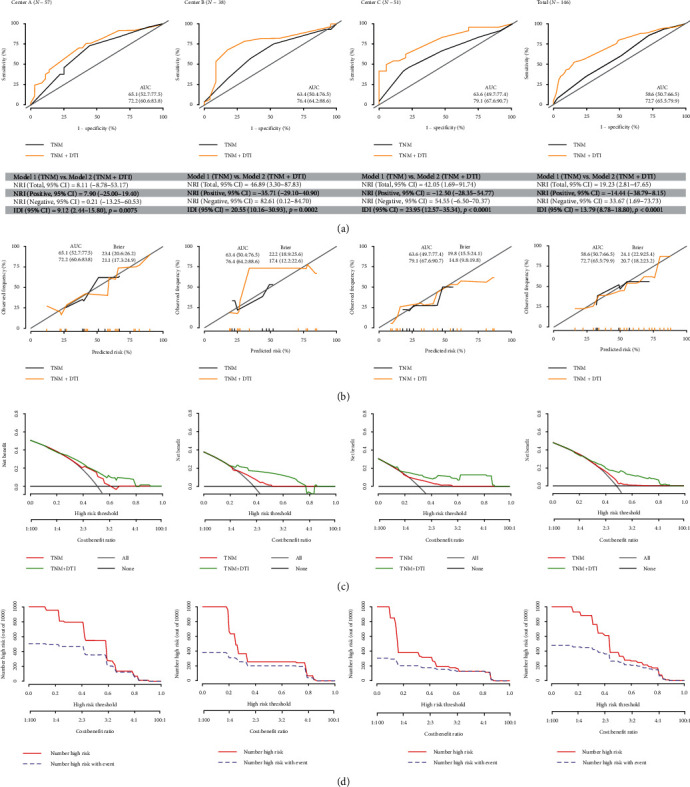
(a) AUC, NRI, and IDI for different models. (b) Calibration curves for different models. (c) Decision curve analysis for different models. (d) Clinical impact curve for Model TNM + DTI.

In Figure 5a, AUC increased from 58.6% to 65.1% to 72.2% to 79.1% after the QA values were incorporated into the regressions. Further, NRI and IDI ranged from 8.11 to 46.89% and 9.12% to 23.95%, respectively. In Figure 5b, lower Brier scores of 14.8–21.1 were recognized in the new prediction model (Model TNM + DTI) compared to 19.8–23.4 in the conventional model (Model TNM), indicating a better fitting. In Figure 5c, higher net benefits were recognized in the Model TNM + DTI compared to Model TNM across different risk thresholds. At last, clinical impact curves for Model TNM + DTI were visualized in Figure 5d.

## 4. Discussion

It is the first study to estimate the longitudinal association between pretreatment brain structural connectivity and 1-year demoralization in Chinese patients with breast cancer using DTI. In the present study, fronto-occipital fasciculus, extreme capsule, and corpus callosum tapetum were recognized as the significant neural pathways associated with 1-year demoralization outcome and these white matter microstructure profiles could provide significant incremental prediction ability to 1-year demoralization.

First, in the current study, CT revealed significant difference in tracking fibers of IFOF at baseline between increased and nonincreased 1-year demoralization groups. Compared to nonincreased groups, a reduction in QA, FA, and AD values was also recognized in patients with increased demoralization. Thus, hypothesis 1 was confirmed. However, IFOF originates from occipital and parietal lobes and terminates in the inferior frontal lobe which are not consistent with previous research where DLPFC and ACC were supposed to be the potential brain areas associated with demoralization [10, 11]. IFOF is a large and complex white matter tract and connects the salience network to the executive control network, while impairments in IFOF can compromise cognitive and linguistic abilities as well as goal-oriented behavior [34, 35]. In addition, the associations between IFOF degeneration and behavioral disorders (i.e., obsessive compulsive disorder) are also confirmed [36, 37]. Thus, we speculate that patients with decreased integrity of IFOF are inherently vulnerable to 1-year increased demoralization before the anticancer treatment and should be followed by clinical interventions (i.e., individualized meaning-centered psychotherapy, IMCP) [15, 16]. Other significant neural pathways such as extreme capsule left, corpus callosum tapetum, and superior longitudinal fasciculus right were also identified based on different anisotropy indicators against the 1-year demoralization outcome. However, due to the small sample size in the current study, high Type I errors should be noted in these potentially false-positive neural pathways although 4,000 permutations as well as a FDR < 0.05 was performed to retain robust tracking fibers. Therefore, these tracking fibers with low proportions should be replicated in future research with a larger sample. In addition, IFOF was only recognized in left hemisphere and this hemispheric disparity may be due to the inherent asymmetry between dominant and nondominant hemisphere which will modulate the brain's response to the traumatic event of a breast cancer diagnosis [38].

Second, the incorporation of QA values into the prediction model achieved NRIs of 8.11%–46.89% and IDIs of 9.12%–23.95%, respectively, confirming hypothesis 2. In addition, the reliability of DTI-based indicators was relatively stable across different centers (AUC = 72.2%–79.1%) confirming QAs in IFOF as a robust predictor for 1-year demoralization, which has also been validated in patient with depression, generalized anxiety, and schizophrenia [39–42]. Based on these findings, in consideration of the noninvasive nature of DTI data collection as well as the increasing access to 3T MRI scanner across different medical centers, it necessitates the development of DTI-based surveillance for high-risk patients with impaired anisotropy in IFOF although a cutoff for the QA values should be further clarified with a larger sample size in breast cancer. The cost of DTI is relatively expensive and the collection of both DTI and fMRI data is recommended at one MRI scanning. However, the coupling among multimodal connectomics is quite complex in the brain network and the association between DTI-and fMRI-based connectivity should be further explored to achieve a better prediction model against demoralization [43, 44]. Also, an additional cost-effectiveness/utilities analysis should be performed to provide health economic data for DTI-based surveillance in breast cancer.

## 5. Limitations

Several issues should be noted in the current study. First, except for age and TNM staging, other confounders such as genetic mutations and socioeconomic status (SES) factors are not controlled in the regressions in consideration of the overfitting issues based on a relatively small sample size. Thus, Type I errors for the significant neural pathways should be considered and a priori anatomical method is recommended to validate these findings with a new patient population. Second, the white matter microstructure may be compromised by chemotherapy, radiotherapy and endocrine-therapy, and our snapshot approach applied in the current study may not capture the dynamic interplay between brain structural connectivity and 1-year demoralization across different treatment phases. Therefore, more longitudinal research should be followed to replicate these findings in this nascent field. Third, researchers suggesting dividing the IFOF into distinct subparts (i.e., superficial, middle, and deep) in consideration of the multiple structural networks involved [45]. In the current study, IFOF was only recognized in left hemisphere and further parcellation-related analysis should be performed to evaluate the prediction ability of subdivisions of the IFOF against demoralization. Fourth, due to the multicenter design, the nuances of technical discrepancies in the collection of DTI imaging and natural variability in tractography should also be taken into consideration. Fifth, as the primary outcome of demoralization was measured in a self-reported manner and the potential effects of social desirability bias should also be noted. At last, measurement errors are not considered in the calculation of changes in demoralization, advanced measurement methods (i.e., cognitive diagnosis models, item response theory) can be explored in future research [46–48].

## 6. Conclusion

Anisotropy in IFOF is a potential prediction neuromarker to 1-year demoralization in patients with newly diagnosed breast cancer.

## Data Availability

The data that support the findings of this study are available on request from the corresponding author. The data are not publicly available due to privacy or ethical restrictions.
